# Gender-Specific Health-Adjusted Life Expectancy of Type 2 Diabetes Mellitus Among the Rural Elderly Population

**DOI:** 10.3389/ijph.2024.1606680

**Published:** 2024-03-13

**Authors:** Yitan Hou, Ze Hu, Feng Jiang, Qiuling Zhao, Chongjian Wang, Yuxiao Zhang

**Affiliations:** ^1^ Department of Social Medicine and Health Management, School of Public Health, Wuhan University, Wuhan, Hubei, China; ^2^ Department of Epidemiology and Biostatistics, College of Public Health, Zhengzhou University, Zhengzhou, Henan, China; ^3^ People’s Hospital of Dongxihu District, Wuhan, Hubei, China

**Keywords:** type 2 diabetes mellitus, health-adjusted life expectancy, life expectancy, sex-specific, elderly population

## Abstract

**Objectives:** This study aimed to estimate the life expectancy (LE) and health-adjusted life expectancy (HALE) of type 2 diabetes mellitus (T2DM) among the rural elderly population.

**Methods:** A total of 10,318 participants aged 65 to 79 were derived from the Henan Rural Cohort. The LE and HALE were calculated via the Sullivan method and multistate life table.

**Results:** Among 10,318 subjects, 1,325 suffered from T2DM at the baseline, and 394 participants had newly-developed T2DM. The results from the Sullivan method showed that the LE, HALE, and HALE/LE were 17.98, 16.18 years, and 89.95% for men aged 65 to 69, and the corresponding estimates for women were 21.81, 18.73 years, and 85.86%, respectively. The LE, HALE and HALE/LE calculated via multistate life table were 19.86, 17.53 years, and 88.29% for men at aged 65, and the corresponding values for women were 25.01, 20.87 years, and 83.44%, respectively.

**Conclusion:** Rural elderly women have a longer LE and HALE of T2DM, but they have lower quality of life than men. More attention should be paid to T2DM among rural elderly people, especially in women.

**Clinical Trial Registration:** The Henan Rural Cohort Study has been registered at Chinese Clinical Trial Register (Registration number: ChiCTR-OOC-15006699). Date of registration: 06 July 2015. http://www.chictr.org.cn/showproj.aspx?proj=11375.

## Introduction

Diabetes mellitus, one of the typical noncommunicable chronic diseases (NCDs), has become an alarming health concern due to its high prevalence and mortality, especially in lower-middle-income countries with inadequate medical service [1, 2]. With the rapid transition of lifestyles, the prevalence of diabetes mellitus has increased significantly in China and has reached epidemic proportions in recent decades [3]. Theoretically, the number of people with diabetes mellitus in China was 35.5 million in 2019 and is estimated to be 54.3 million in 2030 [4]. Although diabetes mellitus was more common in urban areas as several nationwide epidemiological studies illustrated previously, it was associated with greater excess mortality in rural counterparts [3, 5, 6]. In addition, gender difference has played an important role in the occurrence, development, and prevention of type 2 diabetes mellitus (accounting for 90%–95% of all diabetes mellitus) [4, 7, 8].

The rural population is large in China and, compared with their urban counterparts, rural older adults are often faced with a lack of medical care, and the phenomenon of left-behind seniors in rural areas is more likely to lead to a physical health disadvantage in rural older adults. With the acceleration of aging as well as the migrant rural labor force in China, the existing circumstance related to type 2 diabetes mellitus (T2DM) among rural adults aged over 65 has become a health issue worthy of deep research for increasing empty nest elderly in rural areas [9]. Furthermore, the life expectancy (LE) and the health-adjusted life expectancy (HALE) due to T2DM in rural populations aged over 65 was unclear till now. As an index combines morbidity and mortality or other non-fatal health outcome information together, the LE and HALE could better describe the quality of life for the elderly. This study aimed to estimate the LE and HALE due to T2DM for those over 65 years old.

## Methods

### Study Population

Located in central China, Henan Province is a large agricultural province with a total population of 99.37 million in 2020, of which the rural population accounts for 44.57%. Participants in this study were recruited from the Henan Rural Cohort Study, which sampled five rural counties from different geographic regions of Henan Province (southern, central, northern, eastern, and western) through simple cluster sampling. This study contained 39,259 (with a response rate of 93.7%) permanent dwellers aged from 18 to 79 in five regions via multistage stratified cluster sampling. The baseline survey was completed from July 2015 to September 2017 and more details have been described previously [10]. A total of 10,350 individuals aged 65 and older were included in the study. Next, we excluded 2 individuals suffering from type 1 diabetes mellitus (T1DM), 11 previously attacked by gestational diabetes mellitus (GDM), and 19 without information on the assessment of T2DM. Eventually, a total of 10,318 participants were included in the present study to explore the prevalence of T2DM and were further included in the calculation of HALE via the Sullivan method. Among those 10,318 participants, 1,185 of them were lost to follow-up, so only 9,133 participants were included in the calculation of HALE via multistate life table. A flow chart for the inclusion and exclusion of participants in the evaluation of HALE was displayed in the Supplementary Appendix. The study was approved by Zhengzhou University Life Science Ethics Committee, and the written informed consent was obtained from each participant.

### Ascertainment of T2DM

Serum samples, separated from fasting venous blood, were then used to measure participants’ fasting plasma glucose (FPG). In this study, T2DM was defined as FPG ≥7.0 mmol/L or self-reported previously diagnosed by physicians and taking hypoglycemic drugs during the previous 2 weeks except for T1DM and gestational diabetes mellitus [5, 11]. Newly-developed T2DM was defined as a diagnosis of T2DM at follow-up with no diagnosis of T2DM at the baseline.

### Assessment of Covariate Factors

Subjects were divided into three groups by age: 65∼ years, 70∼, years and 75∼ years. Simultaneously, given the relatively lower educational attainment and economic income of the rural elderly, education levels were classified as: illiterate or primary school and above, and per capital monthly income was stratified into three levels: <500, 500∼, or 1,000∼. Moreover, smoking as well as drinking status was categorized as never, former, or current. According to the dietary guide for Chinese residents, high vegetable and fruit intake was considered as a participant consuming more than 500 g of fruits and vegetables per day, and a high fat diet was defined as someone eating meat from livestock or poultry at an average of more than 75 g per day [12]. Body mass index (BMI) was calculated as weight (kg) divided by height (m) squared and was stratified into four levels: underweight (BMI <18.5 kg/m2), normal weight (18.5 kg/m^2^ ≤ BMI <24 kg/m^2^), overweight (24 kg/m^2^ ≤ BMI <28 kg/m^2^), and obese (BMI ≥28 kg/m^2^) [13]. Additionally, according to the International Physical Activity Questionnaire (IPAQ), physical activity was categorized based on the metabolic equivalent of task (MET) per minute per week in low, moderate, and high activity [14].

### HALE Calculation via Sullivan Method

The number of population and deaths derived from the 2017 China Cause-of-death Surveillance Data set was used to compile abridged life tables using Chiang’s method [15] to obtain the estimate of LE. The HALE was further estimated via the Sullivan method combined with LE and the prevalence of T2DM by the following formula [16, 17]:

HALE=1lx∑x=0w1−Pxn×nLx

Where $l_{x}$ is the number of survivors at the exact age *x*; Pxn represents the prevalence of T2DM among individuals with age in the interval (*x, x + n*); Lxn is the number of person-years lived in each age interval; and w represents the maximum age.

### HALE Calculation via Multistate Life Table Method

Combining baseline data with follow-up data, HALE is calculated through the change of T2DM status of subjects during baseline and follow-up investigation, and the transition matrix between states is simulated by the Markov chain. Based on the transition probability between different health states and the construction of a matrix, the multi-state life table method can estimate the life expectancy under different health states. As the multistate life table is based on dynamic tracking data and assumes that different states can be transformed into each other, it is considered to be able to more accurately reflect changes in the health of the elderly population and thus estimate healthy life expectancy more accurately. In the Markov multistate transition model fitted by the “msm” R package [18], three transitions between states were taken into account: 1) from T2DM-free to incident T2DM, 2) from T2DM-free to mortality, and 3) from T2DM to mortality. No reverse transitions were allowed between these states. Then, with the multivariate adjusted model, the R package “ELECT” was applied to calculate total and state-specific life expectancy [19].

### Statistical Analysis

Continuous variables were expressed as means ± SDs and categorical ones as frequencies (proportions). The prevalence of T2DM was presented as a proportion with a 95% confidence interval (CI). Additionally, age-standardized prevalence was calculated by the direct method based on Chinese 2010 census data [20]. Intergroup variances were detected through Student’s t tests and chi-squared tests for continuous variables and categorical ones, respectively.

Life tables via the Sullivan method were conducted in SAS 9.1 software package (SAS Institute, United States) to estimate the LE and HALE. Based on dynamic longitudinal tracking data, the multistate life table was constructed using the ELECT package of R 4.2.2 (R Foundation for Statistical Computing, Vienna, Austria) and then LE and HALE were obtained. Statistical analyses were conducted using SPSS version 21.0 (IBM-SPSS Inc., Armonk, NY), and *p* values in 2-tailed values less than 0.05 were considered to be statistically significant.

## Results

### Demographic Characteristics

Table 1 described the characteristics of participants by gender. A total of 10,318 individuals were absorbed in the present study, including 4,671 (45.27%) men and 5,647 (54.73%) women, and the mean (SD) age of total population was 69.75 (3.78). Compared with men, women were more likely to have such characteristics: widowed/single/divorced/separated, illiterate, never smoking and drinking, higher BMI, and lower physical activity (all *p* < 0.001). However, as for age and *per capita* monthly income, there existed no significance between men and women (*p* > 0.05).

**TABLE 1:** The demographic characteristics of participants (Zhengzhou, China. 2024).

Variable	Total	Men	Women	*p* ^a^
	(n = 10,318)	(n = 4,671)	(n = 5,647)	
Age (years), mean ± SD	69.75 ± 3.78	69.82 ± 3.77	69.70 ± 3.79	0.103
Marital status, n (%)				<0.001
Married/cohabiting	8,140 (78.89)	3,870 (82.85)	4,270 (75.62)	
Widowed/single/divorced/separation	2,178 (21.11)	801 (17.15)	1,377 (24.38)	
Education level, n (%)				<0.001
Illiterate	3,383 (32.79)	826 (17.68)	2,557 (45.28)	
Primary school	4,442 (43.05)	2,060 (44.1)	2,382 (42.18)	
Middle school and above	2,493 (24.16)	1,785 (38.21)	708 (12.54)	
Per capita monthly income (RMB), n (%)				0.372
<500	5,334 (51.70)	2,385 (51.06)	2,949 (52.22)	
500∼	2,957 (28.65)	1,343 (28.75)	1,614 (28.58)	
1,000∼	2,027 (19.65)	943 (20.19)	1,084 (19.20)	
Smoking status, n (%)				<0.001
Never	7,150 (69.30)	1,539 (32.95)	5,611 (99.36)	
Former	1,332 (12.91)	1,321 (28.28)	11 (0.20)	
Current	1,836 (17.79)	1,811 (38.77)	25 (0.44)	
Drinking status, n (%)				<0.001
Never	8,245 (79.91)	2,693 (57.65)	5,552 (98.32)	
Former	713 (6.91)	698 (14.94)	15 (0.27)	
Current	1,360 (13.18)	1,280 (27.41)	80 (1.42)	
Physical activity, n (%)				<0.001
Low	4,414 (42.78)	2038 (43.63)	2,376 (42.07)	
Moderate	3,290 (31.89)	1,224 (26.20)	2066 (36.59)	
High	2,614 (25.33)	1,409 (30.17)	1,205 (21.34)	
Body mass index,^b^ n (%)				<0.001
Underweight	421 (4.10)	217 (4.68)	204 (3.63)	
Normal	4,602 (44.85)	2,337 (50.37)	2,265 (40.30)	
Overweight	3,713 (36.19)	1,569 (33.81)	2,144 (38.15)	
Obese	1,524 (14.86)	517 (11.14)	1,007 (17.92)	
Type 2 diabetes mellitus, n (%)	1,325 (12.84)	481 (10.30)	844 (14.95)	<0.001

Abbreviations: SD, standard deviation.

^a^ *p* was detected the variance between men and women via chi-squared tests or Student’s t-test.

^b^ Variable missing (n = 58).

### Health-Adjusted Life Expectancy via the Sullivan Method

Among a total of 10,318 participants at baseline, 1,325 suffered from T2DM, of which 481 were men and 844 were women. Table 2 displayed the LE, HALE, and the ratio of HALE to LE among participants across ages. Overall, women presented higher LE and HALE than men across all age groups, whereas the trend of the ratio of HALE to LE appeared to be the opposite. As an example, for participants aged 65–69, women showed higher LE (21.81 years) and HALE (18.73 years) than men (LE, 17.98 years; HALE, 16.18 years), whereas the ratio of HALE to LE in women, 85.86%, was lower than that in men (89.95%).

**TABLE 2:** The life expectancy and health adjusted life expectancy via the Sullivan method (Zhengzhou, China. 2024).

Variable	Age group (years)	LE (years)	HALE (years)	HALE/LE (%)
Total	65-	19.78	17.35	87.70
	70-	16.43	14.46	88.00
	75-	13.57	11.98	88.27
Men	65-	17.98	16.18	89.95
	70-	14.88	13.41	90.11
	75-	12.21	11.02	90.27
Women	65-	21.81	18.73	85.86
	70-	18.13	15.64	86.24
	75-	14.98	12.97	86.60

Abbreviations: LE, life expectancy; HALE, health adjusted life expectancy.

### Health-Adjusted Life Expectancy via Multistate Life Table Method

**FIGURE 1:**
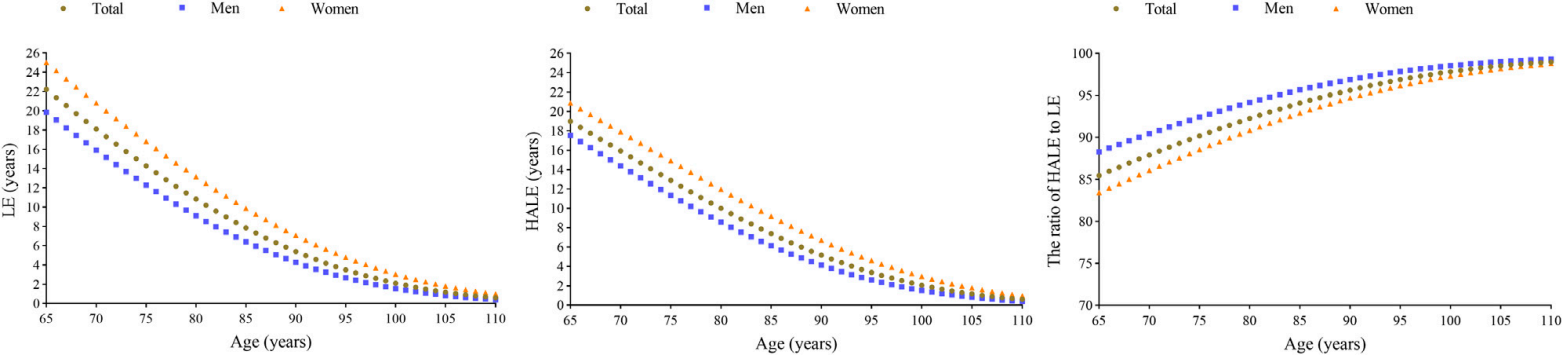
The life expectancy, health adjusted life expectancy, and the ratio of health adjusted life expectancy to life expectancy among the rural elderly population via multistate life table (Zhengzhou, China. 2024). Abbreviations: LE, life expectancy; HALE, health adjusted life expectancy; T2DM, type 2 diabetes mellitus.

During the follow-up period, a total of 394 participants had newly-developed T2DM, of which 163 were men and 231 were women. Similarly with the results calculated via the Sullivan method, both LE and HALE calculated via multistate life table were higher among women than men in the same age group, while the ratio of HALE to LE was lower among women. The HALE due to T2DM of those who were 65 years old was 18.98 years, and was 17.53 and 20.87 years among men and women, respectively. Figure 1 displayed the HALE due to T2DM via multistate life tables. The figure showed that HALE showed a gradual decline trend with age across both men and women. What is more, the gap in HALE between men and women of the same age narrowed with increasing age.

## Discussion

From the large rural sample, the findings revealed that the average LE and HALE for individuals aged 65 to 69, were 19.78 and 17.35 years, respectively, and the ratio of HALE to LE was 87.70%. The LE and HALE showed decreasing trends with increasing age for both genders. Women appeared to have higher LE and HALE compared with men, while the ratio of HALE to LE among women was lower. During the time from baseline to follow-up, 394 participants had newly-developed T2DM, of which163 were men. Similarly with the HALE calculated via the Sullivan method, the HALE calculated via multistate life table also showed a decreasing trend with age, which was higher among women than men at the same age.

Regardless of whether they were calculated via cross-sectional data or longitudinal tracking data, the LE and HALE were higher among women than men of the same age, whereas the ratio of HALE to LE was higher among men than women instead. This finding, which was consistent with other research [21, 22], means that although women live longer than men, they live for longer in unhealthy states, namely, men usually have a higher quality of life than women. Conducted in Barcelona’s general population, a cross-sectional study [23] suggested the difference in HALE between genders was due to inequality in health status rather than differences in reporting. The study also concluded that worse HALE among older women was mainly due to a higher prevalence of disability and chronic conditions [23]. Additionally, women have been reported to be more inclined to suffer from depression and empty-nest syndrome in later life compared with men [24, 25]. Hence, attention should be paid to reducing the prevalence of T2DM among older women and lessening the loss of HALE due to T2DM among older men. Furthermore, the results indicated that interventions should be taken to increase the life expectancy for men in the elderly rural population, while the problem of poor quality of life among women should be emphasized. As the Chinese rural population is rapidly aging due to increased life expectancy and a sharp decline in fertility, it is important to improve the quality of life among older adults in order to cope with this problem. Thus, the findings are meaningful for the formulation of healthy aging policies. In addition, the trend in results calculated by two methods was consistent, although the magnitudes were different. The main reason is that the mortality data of the Sullivan method was obtained from the 2017 China Cause-of-death Surveillance Data set, while the mortality data of the multistate life table method was obtained from the Henan rural cohort study. The multi-state life table method was considered to be more accurate due to the use of longitudinal dynamic data.

This study presented up-to-date information on the gender-specific prevalence of T2DM among adults aged over 65 years old in rural China and explored gender disparities in HALE due to T2DM among this particular population. In addition, to our knowledge, the present study was the first one covering a large sample evaluating the HALE due to T2DM among rural older men and women to clarify the status of life quality of the rural elderly. What is more, the present study contained the calculation of HALE via both the Sullivan method and the multistate life table, which made the results more rigorous and richer. However, some limitations of this study should be noted. First, since the HALE calculated in this study only represents the rural population aged over 65 years old, and the prevalence of T2DM and death data are significantly different between urban and rural populations, a larger sample size of nationwide data is needed to calculate the HALE of the Chinese population. Second, the prevalence of T2DM might be underestimated due to the lack of 2-hour oral glucose-tolerance tests. Third, the Sullivan method of calculating LE and HALE in this study used external data, which may lead to the LE and HALE indicators being relatively underestimated. However, in the study we also used a multistate life table method based on longitudinal data that was able to compensate for this shortcoming. Simultaneously, the HALE also may be overestimated with institutional population uninvolved in the study. However, the questionnaire was concerned with high reliability and validity, and the researchers had been well-trained, so the results in the present study were convincing, even if they were influenced by recall bias. Finally, as the cohort only had one piece of follow-up, the results reported by multistate life tables could be somewhat unstable. In order to make the results more stable and meaningful, more follow-up is needed.

In conclusion, this study showed that T2DM had become a serious public health concern among the older population in rural China. The HALE attributable to T2DM was higher among men compared with women aged over 65, suggesting the life quality of older men tends to be more sensitive to T2DM. Hence, attempts should be made to reduce the prevalence of T2DM and improve life quality among the rural older population, especially among women, which could be a great benefit in boosting the HALE in the entire older population of rural areas.

## Data Availability

The data analyzed during current study are available from the corresponding author on reasonable request.

## Abbreviations

T2DM, Type 2 diabetes mellitus; FPG, Fasting plasma glucose; T1DM, Type 1 diabetes mellitus; LE, Life expectancy; HALE, Health adjusted life expectancy; EQ-5D-5L, EuroqOL-5D-5L; BMI, Body mass index; IPAQ, International Physical Activity Questionnaire; MET, Metabolic equivalent of task; CL, Confidence interval; OR, Odds ratio; SD, Standard deviation; GDM, Gestational diabetes mellitus.
