# Supplementary material for: Gender-Specific Health-Adjusted Life Expectancy of Type 2 Diabetes Mellitus Among the Rural Elderly Population
Source: Int J Public Health. 2024 Mar 13;69:1606680. doi: 10.3389/ijph.2024.1606680 (PMC10965460; doi:10.3389/ijph.2024.1606680)
Supplement: Supplementary file 1 [file DataSheet1.PDF]

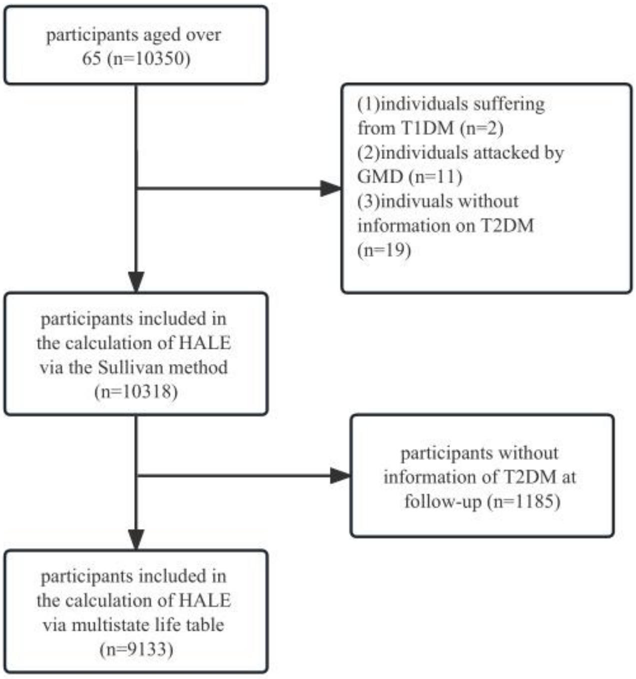

Supplementary Figure 1. The flow chart for the inclusion and exclusion of participants in the evaluation of HALE.
